# Audiological and Vestibular Follow-Up for Children with Congenital Cytomegalovirus Infection: From Current Limitations to Future Directions

**DOI:** 10.3390/children11101211

**Published:** 2024-10-01

**Authors:** Mirko Aldè, Virginia Fancello, Paola Di Mauro, Rachele Canelli, Sandra Zaouche, Chiara Falanga

**Affiliations:** 1 Department of Clinical Sciences and Community Health, University of Milan, 20122 Milan, Italy; 2 Audiology Unit, Department of Specialist Surgical Sciences, Fondazione IRCCS Ca’ Granda Ospedale Maggiore Policlinico, 20122 Milan, Italy; 3 ENT Department, University Hospital of Sassari- Azienda Ospedaliero Universitaria di Sassari, 07100 Sassari, Italy; virginia.fancello@aouss.it; 4 Department of Medical and Surgical Sciences and Advanced Technologies “G.F. Ingrassia” ENT Section, University of Catania, 95123 Catania, Italy; paola.dimauro@unict.it; 5 Otorhinolaryngology Unit, Department of Specialist Surgery, Asl Toscana Centro, 59100 Prato, Italy; rachele.canelli@uslcentro.toscana.it; 6 Department of Otolaryngology, and Otoneurosurgery, Centre Hospitalier Lyon Sud, Hospices Civils de Lyon, 69500 Lyon, France; sandra.zaouche@chu-lyon.fr; 7 Ospedale Cav. R. Apicella, ASL Napoli 3 Sud, 80040 Pollena Trocchia, Italy; chiara.falanga@aslnapoli3sud.it

**Keywords:** congenital cytomegalovirus, hearing loss, children, audiological follow-up, vestibular follow-up

## Abstract

Currently, the guidelines for audiological and vestibular follow-up in children with congenital cytomegalovirus (CMV) are not well-defined. The general recommendation is to evaluate hearing in all children with congenital CMV at the same intervals: once every 3–6 months up to 1 year of age, once every 6 months from 1 to 3 years of age, and once a year from 3 to 6 years of age. Additionally, there are no universally accepted protocols for the vestibular follow-up of children with congenital CMV, although video head impulse test (v-HIT) and cervical vestibular-evoked myogenic potentials (cVEMPs) are sometimes used. This narrative review critically evaluates existing audiological and vestibular follow-up approaches for children with congenital CMV, highlighting the need for personalized protocols. Tailoring follow-up schedules with different timing and methods based on risk factors, such as the trimester of maternal infection, CMV PCR results in amniotic fluid, and valganciclovir use, would indeed allow for more precise evaluations, timely interventions, and optimized resource allocation. This strategy would also alleviate the logistical and emotional burdens on families by ensuring that high-risk children receive more frequent and appropriate assessments and early interventions, while lower-risk children avoid unnecessary testing.

## 1. Introduction

Congenital cytomegalovirus (CMV) infection is an important public health concern, being the most common congenital infection worldwide [1]. CMV is a member of the Her-pesviridae family, and while infections in healthy individuals are often asymptomatic or present with mild flu-like symptoms, congenital infections can result in a range of severe complications [2,3,4]. The majority of children with congenital CMV are asymptomatic at birth, but about 10-15% of them will develop permanent sequelae, the most common being sensorineural hearing loss (SNHL) [5]. Specifically, CMV may affect signaling pathways that are important for the development of peripheral hearing organs [6]. Symptomatic children, who make up about 10% of all congenitally infected newborns, face a higher risk of severe complications, including microcephaly, intracranial calcifications, cerebral hy-poplasia, and various neurodevelopmental disorders. Among these, SNHL stands out as the leading cause of non-genetic hearing loss in children [1]. SNHL can manifest at birth or have a late-onset, be unilateral or bilateral, and range in degree from mild to profound. Audiometric configurations can vary, presenting as flat, sloping, or rising curves. Addi-tionally, hearing thresholds can change over time, showing patterns of stability, fluctua-tion, sudden deterioration, or progressive deterioration [1,7]. Hearing deterioration can occur over months in both asymptomatic and symptomatic congenital CMV infections. However, in symptomatic children, SNHL is typically more severe and tends to progress more rapidly [2,7]. Vestibular loss associated with congenital CMV infection, similar to SNHL, exhibits considerable variability in terms of severity, onset, and progression [2,8]. Neonatal CMV testing, along with genetic screening, should be provided to all children with SNHL to quickly determine the cause and ensure appropriate treatment [9]. A sys-tematic review conducted by Shears et al. revealed that vestibular dysfunction appears more prevalent among children with symptomatic CMV infection, ranging from 22% to 60%, compared to those with asymptomatic CMV, where prevalence rates range from 0% to 12.5% [10]. Despite the clinical significance of CMV-related SNHL and vestibular dys-function, guidelines for audiological and vestibular follow-up in affected children remain inadequately defined. This narrative review aims to provide a comprehensive and critical examination of current approaches to the audiological and vestibular follow-up of chil-dren with congenital CMV infection, highlighting their limitations and exploring future prospects. Titles, abstracts, and full texts from relevant literature were screened to evaluate the content of the studies and extract valuable information. Furthermore, we propose a targeted schedule for monitoring hearing and vestibular functions in children with con-genital CMV, drawing on empirical experience and evidence from the pertinent literature.

## 2. Audiological Follow-Up

### 2.1. Current Audiological Follow-Up Approaches for Children with Congenital Cytomegalovirus Infection

Currently, there are no universally accepted standard guidelines for the long-term audiological follow-up of children with congenital CMV. However, the American Academy of Audiology (AAA) has proposed a specific hearing surveillance model for children affected by congenital CMV, designed to ensure early detection and intervention for any potential hearing problems. The recommended schedule is as follows: hearing assessment every 3–6 months for the first year of life, then every 6 months until 3 years of age, and annually until 6 years of age [11]. This approach is widely adopted by many audiological centers and guidelines from different parts of the world [5,7,12,13,14], as it is recognized for its balance between thorough monitoring and practicality in various healthcare contexts. Other authors have suggested subtle variations in the audiological monitoring schedule for children with CMV infection [15,16,17]. The Joint Committee on Infant Hearing (JCIH) recommends follow-up audiological assessments for infants with congenital CMV no later than 3 months of age and then every 12 months until age 3, or at shorter intervals in response to parental concerns [18]. On the other hand, the International Congenital Cytomegalovirus Recommendations Group, an informal assembly convened during the 5th International Congenital Cytomegalovirus conference in 2015, proposed that audiological follow-up should be performed every 6 months for the first 3 years, and then annually through the adolescent years (ages 10–19) [19]. However, it has been shown that the probability of manifesting delayed SNHL beyond 5 years of age in children asymptomatic for congenital CMV is no different from that of children not infected with the virus [20]. 

Another critical question pertains to the selection of the type of audiological tests for periodic hearing evaluations in children with congenital CMV, as most existing guidelines do not provide specific recommendations on this matter [11,18,19]. Many authors for the audiological follow-up of children with congenital CMV use the clinical auditory brainstem responses (ABRs) with threshold protocol, consisting in click evoked ABRs alone or combined with tone burst evoked ABRs or auditory steady-state response (ASSR) [7,21,22,23,24,25,26,27]. Behavioral audiometry and tympanometry are also often performed [7,21,22,24,27], while the use of otoacoustic emissions (OAEs) and/or automatic brainstem responses (AABR) are generally limited to the first audiological screening and only in some protocols repeated periodically [16,28]. 

### 2.2. Limitations of the Current Audiological Follow-Up Approaches for Children with Congenital Cytomegalovirus Infection

The current hearing surveillance models for children with congenital CMV infection have notable strengths. These include frequent early monitoring, which allows for the prompt detection of any hearing impairments that could interfere with developmental milestones, and long-term follow-up to ensure that any delayed manifestations of SNHL are detected and managed appropriately [2,11,18]. However, these audiological follow-up approaches also have several criticisms and limitations. First, the audiological testing schedules are applied uniformly to all children with congenital CMV, without differentiation in accordance with individual risk factors. This one-size-fits-all approach may not be optimal for every child, particularly those at higher (i.e., children who are severely symptomatic at birth) or lower risk (i.e., children born from mothers who contracted the infection in the third trimester) of developing SNHL [29]. Such a uniform surveillance strategy can indeed lead to either over-monitoring or under-monitoring, neither of which is ideal for optimal patient care.

Another notable issue is that regular monitoring demands considerable healthcare assets, including personnel, equipment, and facility usage. In resource-limited settings, this can lead to significant strain on healthcare systems, making it difficult to maintain consistent follow-up schedules [30,31]. This pressure is exacerbated by the high volume of children requiring follow-up, as CMV is a common congenital infection [32]. Hence, it is essential to prevent superfluous audiological examinations, as these could potentially postpone the follow-up visits for all children who genuinely require them.

Moreover, the burden on families should not be underestimated. Frequent visits to healthcare facilities can be logistically challenging, especially for families living in rural or underserved areas. The financial costs associated with travel, time off work, and potential childcare for siblings can be substantial [33]. These challenges can lead to poor adherence to the follow-up schedule, increasing the risk of missed or delayed diagnoses of SNHL.

In addition to financial and logistical burdens, there are emotional and psychological impacts on families. Repeated medical visits can be stressful for parents, leading to anxiety and affecting overall family well-being [33,34,35]. The constant medical attention can also contribute to a sense of stigma for the child, potentially affecting the overall family. Parents may feel overwhelmed by the constant need for medical appointments and the uncertainty about their child’s future health outcomes [33,34].

Finally, another important limitation lies in the hearing assessment methods used for audiological follow-up. Table 1 and Table S1 shows the different assessments tests that may be used for pediatric audiological follow-up [7,14,16,22,23,24,28,30,36,37,38,39,40,41,42].

Threshold ABR testing typically requires more time than other methods and often necessitates sedation in older children, adding to the complexity and cost of the procedure [38]. Sedation not only increases the risk of potential side effects but also requires additional healthcare resources, such as trained anesthesiologists and monitoring equipment, further burdening the healthcare system. Additionally, the need for sedation can be distressing for parents and caregivers, contributing to their anxiety and concerns about the procedure [43]. Performing threshold ABR testing indiscriminately on all children with congenital CMV can therefore be particularly demanding. 

Alternative methods, such as OAE testing, AABR testing, and age-appropriate behavioral audiometry (behavioral observation audiometry [BOA] for infants up to about 6 months, visual reinforcement audiometry [VRA] for children between about 6 months and 24–30 months, and conditioned play audiometry [CPA] for children between about 24–30 months and 5 years), are quicker but not provide as comprehensive an assessment of the child’s hearing capabilities when used individually compared to threshold ABR testing [36]. Furthermore, AABR requires the child to be asleep and therefore sedation may be necessary [30]. However, combining transiently evoked otoacoustic emission (TEOAE) testing with age-appropriate behavioral audiometry has proven to be a practical and expedient method for initially identifying late-onset SNHL [16]. If hearing deterioration is suspected based on these tests, further evaluation with threshold ABR testing is recommended to confirm and assess the severity of the impairment [30,39].

### 2.3. Future Directions of Audiological Follow-Up Approaches for Children with Congenital Cytomegalovirus Infection

Given the limitations of current hearing surveillance models, it is critical to tailor the audiological follow-up program to individual risk factors. Personalization could significantly enhance the efficiency and effectiveness of monitoring, ensuring that high-risk children receive the attention they need, while low-risk children are not subjected to unnecessary procedures. 

Several factors can be considered when tailoring follow-up schedules. First, it is important to assess the initial hearing status of children with congenital CMV infection. Indeed, patients who already exhibit hearing impairments at birth need close monitoring to track any progression and to initiate early interventions, such as hearing aids or cochlear implants [11,44]. Notably, the ear with poorer hearing tends to deteriorate more rapidly and severely than the ear with better hearing [24]. In addition, SNHL that initially occurs in only one ear is at high risk of evolving into a bilateral condition [11,44,45]. Therefore, in the case of hearing impairment present at birth, regular hearing evaluations are recommended throughout the individual’s lifetime [44]. In particular, the highest risk of hearing deterioration is observed in the first 3 years of life [7,11].

The trimester of maternal infection is another critical factor. Infections occurring in the first trimester are associated with higher risks of severe outcomes, including SNHL, and children may warrant more intensive monitoring [2,46]. In contrast, infections in the second or third trimester are generally associated with lower risks, and these children might not need as frequent follow-ups [46,47]. A comprehensive review by Chatzakis et al. found that the pooled rates of SNHL due to maternal infection during the first, second, and third trimesters are respectively 22.8%, 0.1%, and 0% [48]. In light of these findings, a transdisciplinary group of experts in congenital CMV (European Congenital Cytomegalo-virus Initiative; ECCI) has recently recommended against hearing follow-up in children with established primary maternal infection in the third trimester and normal hearing at birth [44]. On the other hand, no complete consensus has been reached on whether audio-logical follow-up should be performed in children with normal hearing at birth and estab-lished primary maternal infection in the second trimester, as the estimated risk of delayed SNHL is low [44]. As a matter of fact, other authors suggest that hearing follow-up may be recommended only in cases of maternal infection in the first trimester [29,49]. However, it should be considered that, although rarely, second-trimester infection may be associated with delayed-onset sequelae in childhood, mostly partial unilateral SNHL, as reported by the experience of several studies [2,47,50,51,52]. Therefore, it seems useful to ensure early di-agnosis and treatment for these children as well. 

A further factor that may help decide if these children should be regularly tested for hearing could be the CMV PCR in amniotic fluid performed after 17-20 weeks of gestation. Indeed, if a timely PCR test does not detect CMV in the amniotic fluid, it is a strong indi-cator that the babies will not suffer from long-term health complications [44,53]. Con-versely, a recent systematic review and meta-analysis by Gilad et al. found that while the presence of CMV in the amniotic fluid indicates a greater risk for prenatal injury, there is no established link between the viral load in the amniotic fluid and the risk of long-term sequelae in children with congenital CMV [54]. This means that determining the need for future surveillance based on viral load alone would be premature.

The treatment with oral valganciclovir deserves specific considerations. Administra-tion of oral valganciclovir at a dosage of 16 mg/kg/dose twice daily, started within the first month of life and continued for a total duration of 6 months, is recommended for neonates who present moderate or severe symptoms of congenital CMV infection at birth [55]. In-deed, oral valganciclovir, when tolerated, has demonstrated a beneficial impact in both the prevention of SNHL and improvement of hearing threshold in children with sympto-matic congenital CMV infection [2,56,57,58,59]. A growing number of authors also suggest ex-tending the indication for valganciclovir administration to neonates with isolated SNHL at birth due its beneficial effects on hearing outcomes [2,12,27,44,60]. A recent review conducted by Pata et al. revealed that most patients with congenital SNHL showed im-provement or stability in hearing threshold after treatment with oral valganciclovir [59]. Specifically, children with mild or moderate SNHL at birth were more likely to experience improved hearing after treatment, while those with severe or profound SNHL tended to remain stable or worsen [59]. In addition, the incidence of hearing deterioration after treatment with oral valganciclovir in children with normal hearing at birth was found to be exceedingly rare [59].

In this complex scenario, differentiating both the timing of follow-up appointments and the type of audiological testing, contingent upon the risk of developing SNHL, would be beneficial for balancing the costs and effectiveness of the procedures. Indeed, tailoring follow-up schedules to the individual risk factors can help optimize resource allocation, alleviate the logistical and emotional burdens on families, and enhance the sustainability of hearing surveillance in children with congenital CMV infection. 

### 2.4. Proposal for a New Targeted Audiological Follow-Up for Children with Congenital Cytomegalovirus Infection

Based on evidence from the literature and our personal experience, we propose a diversified audiological follow-up schedule tailored to the various risk levels of developing SNHL in children with congenital CMV infection.

For children with congenital CMV and normal hearing at birth (confirmed by the presence of OAE at birth and normal threshold at the conventional ABR assessment performed by the third month of life), the suggested audiological monitoring protocol is as follows:

- Children whose mother was infected in the third trimester (no risk): no regular audiological follow-up is needed. However, immediate hearing evaluations are recommended if SNHL is suspected by parents and the primary care pediatrician, or in case of speech-language delay.
- Children whose mother was infected in the second trimester (low risk): annual audiological assessments using TEOAE and age-appropriate behavioral audiometry until 5 years of age.
- Children with negative amniocentesis (low risk): annual audiological assessments using TEOAE and age-appropriate behavioral audiometry until 5 years of age.
- Children whose mother was infected in the first trimester (high risk): biannual audiological assessments using threshold ABR and age-appropriate behavioral audiometry for the first 3 years of life, and then annual audiological assessments using TEOAE and age-appropriate behavioral audiometry from 3 to 5 years of age.
- Children with positive amniocentesis (high risk): biannual audiological assessments using threshold ABR and age-appropriate behavioral audiometry for the first 3 years of life, and then annual audiological assessments using TEOAE and age-appropriate behavioral audiometry from 3 to 5 years of age.
- Children treated with valganciclovir for 6 months started within the first month of life: audiological assessments using threshold ABR immediately after the 6-month treatment. Subsequently, if a normal threshold is found, audiological follow-up using TEOAE and age-appropriate behavioral audiometry at the same frequency as the program based on previous risk factors (trimester of maternal infection and amniocentesis results).

If the maternal trimester of infection and the results of amniocentesis are unknown, children should be considered to be at high risk of developing SNHL. Further audiological assessments are recommended if parents and the primary care pediatrician suspect the presence of new-onset SNHL.

Hearing evaluations should be conducted in the absence of outer or middle ear infections (confirmed by otomicroscopy and tympanometry), as they could affect the results of TEOAE and age-appropriate behavioral audiometry.

If TEOAE and age-appropriate behavioral audiometry are suggestive of hearing deterioration, a threshold ABR should be performed as soon as possible.

All children who receive a diagnosis of SNHL confirmed by threshold ABR should undergo regular audiological assessments: the frequency of follow-up should be tailored to each child, with age-appropriate behavioral audiometry and threshold ABR performed at least twice a year until the child is able to perform a pure-tone audiometry.

## 3. Vestibular Follow-Up

### 3.1. Current Vestibular Assessment Approaches for Children with Congenital Cytomegalovirus Infection

An increasing body of research suggests that including vestibular assessment in the follow-up of children with congenital CMV is crucial, as the impact of vestibular and bal-ance disorders in this clinical population is not negligible [2,7,8,9,10,11,29,44,61,62,63,64,65]. However, currently there is no standardized vestibular monitoring protocol for children with con-genital CMV infection, and knowledge on the topic remains limited, as most studies in the literature are characterized by the use of various quantitative vestibular tests, small sam-ple sizes, lack of systematic screening, and short-term follow-up [10,44,62,63]. This may explain the high variability in the prevalence of reported vestibular and balance disorders in children with congenital CMV, ranging from 14% to 90.4% and 6 to 50%, respectively [8,61,66,67]. Interestingly, Chebib et al. showed the vestibular part of the inner ear is sig-nificantly more frequently impaired than the cochlear part [68]. Moreover, according to Shears et al., any child who presents with vestibular, balance or developmental abnormal-ities should be tested for congenital CMV using stored dried blood spots samples [10]; in-deed, this method enables the diagnosis of congenital CMV infection with high sensitivity, even after the first three weeks of life [69,70]. After all, CMV has a demonstrated tropism for the vestibular part of the inner ear, with cytomegalic cells found in both the semicircu-lar canals and otolith organs (utricle and saccule) [62,71]. Therefore, during vestibular evaluations in children with congenital CMV infection, it is paramount to investigate both canal and otolith functions. 

Table 2 and Table S2 shows the different assessments tests that may be used for pediatric vestibular follow-up [72,73,74,75,76,77,78,79,80,81].

For younger children (under the age of 3-4 years), the video head impulse test (v-HIT) and cervical vestibular-evoked myogenic potentials (cVEMPs) have been suggested as the most feasible vestibular assessments [8,64,75,80,81,82,83]. The v-HIT is used to evaluate the function of the semicircular canals, while cVEMPs are mainly employed to measure the function of the saccule and the inferior branch of the vestibular nerve; these tests provide site of lesion vestibular information in a brief time (usually less than 20 min), making them effective as screening tools in early childhood [8,74,80,81,83].

In pediatric individuals, the v-HIT is primarily conducted for the examination of horizon-tal semicircular canals, with evaluations of the vertical semicircular canals generally in-cluded for older and more compliant children [64,77,80]. From about age 3 years, ocular vestibular-evoked myogenic potentials (oVEMPs), which assess the function of the utricle and the superior branch of the vestibular nerve, are also sometimes performed [64,76,80]. 

Another valuable vestibular test for pediatric patients is rotary chair testing, which mainly evaluates the function of the horizontal semicircular canal and the superior branch of the vestibular nerve; it has been proven to have a major sensitivity compared with v-HIT in detecting unilateral and bilateral vestibular losses [84,85]. However, while highly effective, rotary chair testing needs expensive equipment and typically requires more time compared to other assessments, limiting its use for screening purposes [75,85]. It is important to note that the exact duration of various testing procedures can vary based on individual circumstances, including the child’s age, level of cooperation, and any ad-ditional time needed for explanation, setup, and breaks [75]. 

Although caloric testing has traditionally been considered the gold standard for evaluating horizontal canal function, it is currently reserved for older children and is not the standard method for initial vestibular assessments in pediatric patients due to its in-vasive nature [75,84].

A practical approach to assess balance is posturography, which provides various objective measures, such as the quantification of body sway, through static and dynamic force platforms. This technique is especially valuable in evaluating balance in older chil-dren who have the ability to follow instructions and remain still during the test [72,86]. The insights gained from posturography are particularly useful for designing effective treatment plans and monitoring progress in CMV-infected children with balance disor-ders [7,62,67].

Nowadays, the application of v-HIT is spreading among pediatric patients [8,64,75,80,83]. However, while the v-HIT offers a quantitative measure of Ves-tibulo-Ocular Reflex (VOR) gains, it does not evaluate the functional implications of ves-tibular deficits, possibly underestimating their impact on children’s lives. To bridge this gap, the conventional v-HIT can be supplemented with the functional head impulse test (f-HIT), an innovative tool that evaluates the integration between the cerebellar, cerebral, vestibular, and visual systems. This test specifically assesses a patient’s ability to recog-nize a rapidly appearing and differently oriented Landolt C optotype on a screen during passive head accelerations in the horizontal or in vertical planes [87]. There is no strict age limit for performing the f-HIT, but it is generally conducted in children older than 6 years, as it may be challenging to get very young patients to cooperate [78,88].

It is noteworthy that, just as with SNHL, vestibular and balance disorders in children with congenital CMV can appear regardless of whether they displayed symptoms at birth [7,10,62,63,67]. These dysfunctions can either be present from birth or emerge later in life, as well as worsen over time [8,10,61,64,82]. As a result, due to the potential for late-onset and progression of disorders, it is recommended that children with congenital CMV infec-tions undergo regular vestibular and balance evaluations [2,8,10,62,63,64,65,82]. Some authors have found that CMV-infected children with SNHL have a higher prevalence of vestibular and balance disorders compared to those with normal hearing [62,63,64,82]. This observa-tion suggests that SNHL and vestibular impairments might be influenced by similar risk factors, as also corroborated by two studies conducted by Dhondt al., who demonstrated a higher prevalence of vestibular loss in children with pathological magnetic resonance imaging (MRI) or whose mother had seroconverted in the first trimester [64,82]. Therefore, pediatric patients with congenital CMV infection who are at a higher risk of developing SNHL should undergo more frequent monitoring, including not only rigorous audiologi-cal assessments but also vestibular evaluations [44,62,63,64,82]..

### 3.2. Proposal for a New Targeted Vestibular Follow-Up for Children with Congenital Cytomegalovirus Infection

Taking into account the evidence of the current literature, and drawing from our experience, we propose the following vestibular and balance monitoring protocol for pediatric patients with congenital CMV and normal vestibular function at baseline (confirmed by cVEMPs and v-HIT of the horizontal canals performed within the first 6 months of life):

- Children whose mother was infected in the third trimester (no risk): no regular vestibular follow-up is needed. However, immediate vestibular evaluations are recommended if vestibular loss is suspected by parents and the primary care pediatrician, or in case of abnormal motor development.
- Children whose mother was infected in the second trimester, with negative amniocentesis, normal hearing and no pathological lesions on brain MRI: annual vestibular assessments using cVEMPs and v-HIT of the horizontal canals until the age of 5 years, including oVEMPs and v-HIT of the vertical canals at least once after the third year of age.
- Children whose mother was infected in the first trimester and/or with positive amniocentesis and/or diagnosed with SNHL and/or with pathological lesions on brain MRI (high risk): biannual vestibular assessments using cVEMPs and v-HIT of the horizontal canals for the first 3 years of life, and then annually with addition of oVEMPs and v-HIT of the vertical canals until the age of 5 years.

Posturography should also be performed at least once by the end of the five-year follow-up for all children with congenital CMV infection. In addition, the wider use of f-HIT in the near future could play a significant role in improving the identification and management of vestibular disorders.

In cases where the trimester of maternal infection and the results of the amniocentesis are not known, it is prudent to regard these children as having a high risk for vestibular loss.

Moreover, if parents or the primary care pediatrician observe signs of abnormal motor development in a child with congenital CMV, it is advisable to conduct additional vestibular assessments.

Strict compliance with vestibular follow-ups is indispensable, as diagnosing vestibular impairment in pediatric patients often presents complexities and risks of underestimation. Children may have difficulty expressing their symptoms accurately, and their brains can adapt to vestibular deficits, masking the condition and hindering early detection [10,89]. It is important to recognize that conducting vestibular tests in the pediatric population can be particularly challenging. Beyond issues of children’s limited attention and cooperation, additional comorbidities, such as severe neurological complications, can further hinder these procedures. Therefore, it is essential to have a specialized team of qualified professionals, including audiovestibular physicians and audiologists, who are adept at performing and interpreting these tests and have experience working with children, whether or not they have neurological disabilities. Such expertise is crucial for ensuring the accuracy and reliability of the tests. In any case, routine follow-up vestibular tests, such as the v-HIT of the lateral canals and cVEMPs, should be a core competency of all pediatric tertiary level audiology centers. These centers should indeed be equipped to provide comprehensive audiological and vestibular assessments for all children with congenital CMV infection.

## 4. Rehabilitation Strategies for Children with Hearing and/or Vestibular Impairments

### 4.1. Hearing Rehabilitation

The primary purpose of conducting accurate long-term audiological and vestibular fol-low-ups is to promptly detect any deterioration in hearing and vestibular functions. This approach helps mitigate the risk of leaving SNHL and vestibular deficits unaddressed.

Untreated SNHL is known to have significant adverse effects, not only escalating societal costs but also hampering essential aspects of a child’s development, such as language acquisition, attention, behavior, emotional health, and overall quality of life [2,11]. For children diagnosed with SNHL, it is imperative to promptly fit appropriate hearing aids [7]. However, in cases of severe to profound SNHL, hearing aids may not provide ade-quate rehabilitation, necessitating the consideration of cochlear implants [2,11]. Cochlear implants in children with congenital CMV generally yield positive long-term language outcomes, although effectiveness may be diminished in those with severe neurological sequelae [2]. Clinicians play a crucial role in managing expectations and providing com-prehensive care, ensuring that parents are well-informed about the potential delays and challenges in the rehabilitation process, which may require intensive speech therapy after surgery [90]. The application of cochlear implants even in children with single-sided deafness (SSD) has emerged as a significant advancement in pediatric audiology [91]. While the FDA (Food and Drug Administration) recently approved cochlear implants for children aged 5 and older with SSD—starting with MED-EL in 2019 and followed by Cochlear Americas in 2022—clinical practice increasingly supports earlier unilateral cochlear implantation, especially in children with congenital CMV [91,92]. By providing timely auditory stimulation to the SSD ear, the brain’s ability to integrate sounds from both ears is enhanced, leading to improved spatial hearing and sound localization. This is particularly crucial for children, as their auditory pathways are still maturing, and early intervention can support more natural language acquisition and cognitive development [92]. Furthermore, early cochlear implantation in the SSD ear can act as a safeguard against the potential rapid deterioration of hearing in the contralateral ear, which is a se-rious concern in children with congenital CMV [2,91,92]. Additionally, this approach can significantly enhance auditory sensitivity in children with severe visual impairments, such as CMV-related chorioretinitis, thereby improving overall sensory input [2]. There-fore, implanting the SSD ear early, rather than delaying intervention, is advantageous in maximizing auditory and neurodevelopmental outcomes in children with congenital CMV [2,91]. However, it is important to remember that patients with cochlear implants need special attention to their vestibular function, as it can either remain unchanged, de-teriorate temporarily or permanently, or even improve following implantation surgery [93,94]. Recent evidence suggests that robotic-assisted cochlear implantation, with its pre-cision and minimally invasive approach, may offer better preservation of vestibular func-tion compared to traditional methods [95].

### 4.2. Vestibular Rehabilitation

All children with vestibular and balance disorders should be referred to specialized facili-ties capable of providing a targeted rehabilitation program. While strategies for the reha-bilitation of SNHL are increasingly well-established and show evident long-term results in the pediatric population, there is less experience with the rehabilitation of children’s vestibular loss, both in terms of approaches and outcomes [96]. As a matter of fact, reha-bilitation for vestibular loss in children is particularly demanding, given the need for en-gagement and comprehension of therapeutic exercises, which can be difficult to achieve with very young children or those with neurological disabilities [96,97]. Vestibular reha-bilitation for children is designed to promote adaptation, habituation, and substitution to compensate for vestibular deficits [97,98]: (1) Adaptation exercises focus on improving gaze stability and balance; (2) Habituation exercises aim to reduce vertigo and dizziness by gradually exposing the child to movements that provoke symptoms; (3) Substitution strat-egies involve teaching the child to rely more on visual and proprioceptive cues to com-pensate for the lack of vestibular input.

Children whose vestibular dysfunction leads to functional disorders should undergo tar-geted vestibular rehabilitation tailored to their age and specific needs [97,98]. Early inter-vention is crucial, especially in cases of congenital vestibular dysfunction, where training should focus on developing various aspects of postural control, including anticipatory and reactive postural adjustments [97,98]. While gaze stabilization and balance exercises are beneficial, certain methods like optokinetic stimulation and virtual reality are not recommended for young children due to their limited efficacy and the current lack of evi-dence supporting their use [98]. On the other hand, traditional exercises might not be ef-fective if they do not capture the child’s interest or align with their developmental stage. The success of vestibular rehabilitation in children hinges on the involvement of specifi-cally trained professionals who can integrate playful and interactive activities, making the therapy both engaging and effective [97]. Indeed, although vestibular rehabilitation in children is not widely practiced, specialized centers with qualified personnel have re-ported positive long-term outcomes, including improved postural control, enhanced gross motor skills, and resolution of symptoms such as dizziness and imbalance [96,97,98]. 

Finally, it is important to acknowledge that, compared to profound bilateral SNHL, the options for restoring vestibular functions in children with complete bilateral vestibular loss are considerably more limited [94,99]. Unlike cochlear implants, which have become a routine procedure for hearing restoration, vestibular implants are not yet standard prac-tice, especially in pediatric populations [99]. In fact, vestibular implants, which use a pro-cessor to convert motion data into electrical signals that stimulate the vestibular nerve, are still solely in the research phase for children [99,100]. Initial studies in adults have shown promise, offering an additional tool for managing balance and spatial orientation [99,100]. Vestibular implants could also act as a “vestibular pacemaker” for individuals with fluc-tuating vestibular function [99]. The potential of these implants to complement traditional vestibular rehabilitation in cases where conventional methods may fall short is signifi-cant, but more research is needed to fully understand their long-term efficacy and safety in younger patients.

## 5. Conclusions

Congenital CMV infection presents a complex public health issue, particularly due to its potential to induce late-onset SNHL and vestibular dysfunction in affected children. While current follow-up approaches are beneficial, they often fail to account for individual risk factors, leading to either excessive or insufficient monitoring. Hence, it is essential to prioritize the differentiation in the frequency and type of audiological and vestibular assessments, based on each child’s individual risk of developing SNHL and vestibular loss. For instance, children whose mothers were infected in the third trimester may not require regular follow-ups, whereas those infected in the first trimester or with positive amniocentesis results need closer monitoring. 

Personalizing the audiological and vestibular follow-up approaches not only may ensure timely detection and intervention for potential hearing and vestibular deterioration, but also helps to alleviate the logistical, emotional, and financial burdens associated with frequent assessments.

Future researches and collaborations among healthcare professionals will be crucial in refining audiological and vestibular follow-up protocols for children with congenital CMV infection across various healthcare settings.

## Figures and Tables

**Table 1:** Assessment tests for pediatric audiological follow-up.

Audiological Test	Age Range (Approximately)	What It Studies	Limitations	Test Duration
Behavioral Observation Audiometry (BOA)	<6 months	Behavioral responses to sound stimuli (e.g., startle reflex, eye-widening, or changes in sucking patterns).	Depends on observer’s interpretation; influenced by infant’s state (e.g., sleep, hunger); cannot quantify degree of hearing loss.	Variable (generally 10–20 min)
Visual Reinforcement Audiometry (VRA)	6 months to 24–30 months	Behavioral responses to sound stimuli (child turns head toward sound source).	Depends on the child’s cooperation and ability to focus on the visual reinforcers; quick habituation can decrease response rate; may not be feasible for children with developmental disabilities.	Variable (generally 10–20 min).
Conditioned Play Audiometry (CPA)	24–30 months to 5 years	Behavioral responses to sound stimuli through a play activity are recorded to determine hearing thresholds at various frequencies.	Requires the child’s cooperation and attention; may not be possible for children with developmental disabilities.	Variable (generally 15–30 min).
Pure-Tone Audiometry (PTA)	≥5 years	Hearing thresholds at various frequencies (audiogram) using AC and BC to identify the softest sound a person can hear.	Requires consistent child cooperation and response; not suitable for children with cognitive impairments; may miss auditory processing disorders.	Variable (generally 15–30 min).
Otoacoustic Emissions (OAEs)	All ages	Evaluates cochlear (outer hair cell) function by measuring sounds generated by the inner ear in response to auditory stimuli; can determine the presence of emissions typically indicating normal hearing thresholds up to approximately 30–40 dB HL.	Does not assess beyond cochlea; affected by outer or middle ear conditions (e.g., wax, fluid); provides a pass/fail result without detailed threshold information; can give a false positive in presence of outer/middle ear pathology, or a false negative (missing mild hearing loss); can be affected by the presence of noise or patient movement; cannot detect auditory neuropathy or retrocochlear pathologies.	Variable (generally 5–10 min).
Automated Auditory Brainstem Responses (AABRs)	All ages	Screens auditory nerve and brainstem function by detecting electrical activity in response to sound; the test automatically analyzes the responses to determine if they are present or absent, providing a pass/fail result for hearing screening.	Provides a pass/fail result without detailed threshold information; may miss mild hearing loss; cannot differentiate between types of hearing loss (e.g., sensorineural vs. conductive); requires a quiet and still environment; necessitates natural sleep or often sedation in older children.	Variable (generally 5–15 min).
Threshold Auditory Brainstem Responses (ABRs)	All ages	Measures electrical activity from auditory nerve and brainstem in response to sound at different frequencies (click-evoked ABR for medium/high frequencies [2–4 kHz] and tone burst-evoked ABR for low frequencies [250–500 Hz] and intensity levels to determine hearing thresholds.	Time-consuming; requires a quiet and still environment; necessitates natural sleep or often sedation in older children.	Variable (generally 30–90 min).
Auditory Steady-State Responses (ASSR)	All ages	Measures frequency-specific auditory thresholds using continuous modulated tones; predicts hearing thresholds in very young children at lower frequencies (including 0.5 kHz).	Time-consuming; necessitates natural sleep or often sedation in older children; may be less effective in cases of auditory neuropathy; results can be influenced by neurological conditions; less effective at very high frequencies.	Variable (generally 30–90 min).

ABR = Auditory Brainstem Response; AC = Air Conduction; BC = Bone Conduction.

**Table 2:** Assessment tests for pediatric vestibular follow-up.

Vestibular Test	Age Range (Approximately)	What It Studies	Limitations	Test Duration
Cervical Vestibular-Evoked Myogenic Potentials (cVEMPs)	>2 months	Evaluates the saccule and inferior vestibular nerve (and also utricle through BC).	AC cVEMPs are often absent in the presence of middle ear pathologies; maintaining SCM contraction may be difficult; the test may not be reliable in patients with neck problems.	Variable (generally 15–20 min)
Video Head Impulse Test (v-HIT) of Lateral Canals	≥3 months	Measures the gain of the VOR and the presence of corrective saccades specific to lateral semicircular canals; assesses the superior branch of the vestibular nerve.	Tightly fitting goggles may be uncomfortable for children; artifacts can affect accuracy, especially in uncooperative children (requires their attention); the test does not assess functional implications of vestibular deficits.	Variable (generally 10–15 min)
Rotary Chair Testing	>6 months	Evaluates the overall function of the vestibular system by assessing the VOR gain, phase, and symmetry.	Goggles may be uncomfortable for children; rotary chair gain is affected by the child’s attention; children often sit on their parent’s lap during testing, which can artificially inflate rotary chair gain.	Variable (generally < 15 min)
Ocular Vestibular-Evoked Myogenic Potentials (oVEMPs)	>2 years	Evaluates the utricle and the superior vestibular nerve.	Children might struggle to maintain an up-gaze position; AC oVEMPs are often absent in the presence of middle ear pathologies.	Variable (generally 15–20 min)
Video Head Impulse Test (v-HIT) of Vertical Canals	>2 years	Measures the gain of the VOR and the presence of corrective saccades specific to vertical semicircular canals; assesses the superior (anterior semicircular canals) and the inferior branch (posterior semicircular canals) of the vestibular nerve.	Same limitations as v-HIT for lateral canals and also requires more cooperation from children.	Variable (generally 10–15 min)
Posturography	>5 years	Analyzes balance control and postural stability.	Limited in diagnosing specific vestibular disorders; influenced by other factors affecting balance, such as proprioception and vision; younger children may have difficulty following instructions.	Variable (generally 20–30 min)
Functional Head Impulse Test (f-HIT)	>5 years	Evaluates functional impact of vestibular disorders on gaze stability.	Requires high patient cooperation and quick head movements; limited normative data for younger children.	Variable (generally 15–20 min)
Caloric testing	>5 years	Evaluates the horizontal canal and superior branch of the vestibular nerve.	Uncomfortable for patients; time-consuming; can cause dizziness, nausea, and discomfort; younger children may find it particularly distressing.	Variable (generally 20–25 min)

AC = Air Conduction; BC = Bone Conduction; cVEMPs = Cervical Vestibular-Evoked Myogenic Potentials; oVEMPs = Ocular Vestibular-Evoked Myogenic Potentials; SCM = sternocleidomastoid; v-HIT = Video Head Impulse Test; VOR = Vestibulo-Ocular Reflex.

## Data Availability

Not applicable.

## Supplementary Materials

The following supporting information can be downloaded at: https://www.mdpi.com/article/10.3390/children11101211/s1, Table S1: Description of how audiological tests are performed in children; Table S2: Description of how vestibular tests are performed in children.
