# Supplementary material for: Audiological and Vestibular Follow-Up for Children with Congenital Cytomegalovirus Infection: From Current Limitations to Future Directions
Source: Children (Basel). 2024 Oct 1;11(10):1211. doi: 10.3390/children11101211 (PMC11506510; doi:10.3390/children11101211)
Supplement: Supplementary file 1 [file children-11-01211-s001.zip › children-3174311-supplementary.pdf]

Supplementary Table S1. Description of how audiological tests are performed in children.

Audiological Test	Procedure
**Behavioral Observation Audiometry (BOA)**	The infant is placed in a soundproof room, usually on a parent's lap. Various sound stimuli are presented through speakers or other noisemakers.
**Visual Reinforcement Audiometry (VRA)**	The child is seated in a soundproof room, often on a parent's lap. Sound stimuli are presented through speakers or other noisemakers, and the child is trained to associate the sound with a visual reward (e.g., a lighted toy or animated video).
**Conditioned Play Audiometry (CPA)**	The child is seated in a soundproof room, and is trained to perform a play activity (e.g., placing a block in a bucket or a ring on a stick) whenever he/she hears a sound. The sounds are presented through speakers or headphones.
**Pure-Tone Audiometry (PTA)**	The child is seated in a soundproof room and wears headphones. Pure tones at various frequencies and intensities are presented to one ear at a time. The child is instructed to signal (e.g., raising a hand or pressing a button) whenever he/she hears a sound. Both AC (through headphones) and BC (through a bone vibrator placed on the mastoid bone) tests are conducted.
**Otoacoustic Emissions (OAEs)**	The child is seated or lying down in a quiet environment. A small probe, which contains both a speaker and a microphone, is placed in the ear canal. The speaker delivers a series of stimuli at specific frequencies and the microphone measures the sounds that the cochlea (specifically the outer hair cells) produce in response to these stimuli.
**Automated Auditory Brainstem Responses (AABRs)**	The child is usually asleep or sedated in a quiet environment. Electrodes are placed on the scalp at the following locations: the forehead, the vertex, and the mastoid or earlobe. Insert earphones deliver a series of clicks at a fixed intensity (typically 35 dB nHL).
**Threshold Auditory Brainstem Responses (ABRs)**	The child is typically asleep or sedated in a quiet environment. Electrodes are placed on the scalp at the following locations: the forehead, the vertex, and the mastoid or earlobe of the test ear. Insert earphones are used to deliver click or tone burst stimuli at varying frequencies and intensity levels.
**Auditory Steady-State Responses (ASSR)**	The child is usually asleep or sedated in a quiet environment. Electrodes are placed on the scalp at the following locations: the forehead, the vertex, and the mastoid or earlobe of both ears. Insert earphones deliver continuous modulated tones at multiple frequencies and intensity levels.

AC = Air Conduction; BC = Bone Conduction.

Supplementary Table S2. Description of how vestibular tests are performed in children.

Vestibular Test	Procedure
**Cervical Vestibular-Evoked Myogenic Potentials (cVEMPs)**	The child is generally positioned in a sitting position, often on a parent's lap (or lying supine for older children). Electrodes are placed as follows:<br>• Active Electrode: on the superior/middle third of the SCM muscle on one side of the neck.<br>• Reference Electrode: on the sternoclavicular junction.<br>• Ground Electrode: on the forehead.<br>The child is encouraged to maintain SCM muscle contraction by searching for a toy/cartoon presented on the opposite side of the acoustic stimulus. The stimulus is delivered either through AC using a speaker or through BC using a vibrator. It is an inhibitory response recorded from the ipsilateral SCM muscle.
**Video Head Impulse Test (v-HIT) of Lateral Canals**	The child wears tightly fitting goggles with a high-speed camera. The examiner rapidly moves the child's head in small, quick turns while the child focuses on an attractive target (i.e., toys; flashing lights). For children under the age of 3, a remote video method should be used.
**Rotary Chair Testing**	The child is seated in a rotary chair, often on a parent's lap. The chair rotates at various speeds while the child's eye movements are recorded using video goggles.
**Ocular Vestibular-Evoked Myogenic Potentials (oVEMPs)**	The child is generally positioned in a sitting position, often on a parent's lap (or lying supine for older children). Electrodes are placed as follows:<br>• Active Electrode: beneath each lower eyelid.<br>• Reference Electrode: about 1 cm below the active electrode.<br>• Ground Electrode: on the forehead.<br>The child is encouraged to look upward at an attractive visual target, usually at an angle of about 30 degrees above the horizontal plane, to elicit oVEMP responses by AC or BC acoustic stimulations. It is an excitatory response recorded from the inferior oblique muscle on the side contralateral to stimulation.
**Video Head Impulse Test (v-HIT) of Vertical Canals**	Similar to v-HIT for lateral canals, but the head movements are directed in the planes of the anterior and posterior semicircular canals.
**Posturography**	The child stands on a platform that may move or remain stationary while visual and/or proprioceptive cues are altered. The balance responses are measured.
**Functional Head Impulse Test (f-HIT)**	The child is seated and asked to recognize the orientation of the Landolt C optotype on a screen while the head is moved rapidly by the examiner.
**Caloric testing**	Warm or cold water or air is irrigated into the ear canal, one ear at a time, while the child lies down with his/her head elevated. Eye movements (nystagmus) are recorded to evaluate the vestibular response.

AC = Air Conduction; BC = Bone Conduction; SCM = sternocleidomastoid
